# Real-World Safety Profile of Biologics Used in Rheumatology: A Six-Year Observational Pharmacovigilance Study in the Calabria Region

**DOI:** 10.3390/pharmaceutics14112328

**Published:** 2022-10-28

**Authors:** Agnese Gagliardi, Francesco Salvatore Iaquinta, Rosa Daniela Grembiale, Caterina De Sarro, Antonio Fabiano, Domenico Fraija, Caterina Palleria, Rossella Romeo, Adele Emanuela De Francesco, Maria Diana Naturale, Rita Citraro, Luca Gallelli, Antonio Leo, Giovambattista De Sarro

**Affiliations:** 1 Department of Health Sciences, School of Medicine and Surgery, Magna Graecia University of Catanzaro, 88100 Catanzaro, Italy; 2 UOC Pharmacy, “Mater Domini” Hospital, 88100 Catanzaro, Italy; 3 System and Applied Pharmacology@University Magna Grecia (FAS@UMG) Research Center, Science of Health Department, School of Medicine, Magna Graecia University of Catanzaro, 88100 Catanzaro, Italy

**Keywords:** biological drugs, adverse events (AEs), pharmacovigilance

## Abstract

**Background:** The introduction of biological agents into the clinical armamentarium has modified the management of moderate-severe inflammatory arthritis (IA). However, these drugs can lead to serious adverse events (SAEs) and unpredictable adverse events (AEs) that are difficult to detect in pre-marketing clinical trials. This pharmacovigilance project aimed to study the AEs associated with biologics use in rheumatology. **Methods:** The current investigation is a multicenter, prospective, observational cohort study based on the Calabria Biologics Pharmacovigilance Program. Patients treated with one biologic agent from January 2016 to January 2022 were enrolled. **Results:** Overall, 729 (86.3%) of a total of 872 patients did not develop AEs or SAEs, whereas 143 (16.4%) patients experienced at least one AE, of which 16 (1.8%) had at least one SAE. The most common AEs were administration site conditions followed by gastrointestinal, nervous system and skin disorders. We reported a total of 173 switches and 156 swaps. Switches mainly occurred for inefficacy (136; 77.7%), whereas only 39 (22.3%) were due to the onset of an AE. Primary/secondary failure was the most frequent reason for swaps (124, 79%), while AEs onset led to 33 (21%) swaps. **Conclusions:** This study supports the validity of our program in monitoring and detecting AEs in the rheumatological area, confirming the positive beneficial/risk ratio of biologics.

## 1. Introduction

The term “inflammatory arthritis” refers to a group of diseases, including rheumatoid arthritis (RA), psoriatic arthritis (PsA), and ankylosing spondylitis (AS), that share clinical characteristics, such as those of an inflammatory and chronic nature triggered by an overactive immune system [1]. These conditions are all characterized by joint pain, which, when untreated, can result in a severe loss of joint function and a decline in the patients’ quality of life [2]. Currently, the incidence of arthritis is higher in younger people, above all if overweight or obese, but it is also quite common in the middle-aged and elderly populations [3]. 

Conventionally, the most common anti-arthritis therapeutic approaches are classified into three types: nonsteroidal anti-inflammatory drugs (NSAIDs), conventional synthetic disease-modifying anti-rheumatic drugs (csDMARDs), and biological agents [4,5]. NSAIDs and glucocorticoids were previously the only available therapeutic option for inflammatory arthritis to control pain and inflammation while also preventing long-term joint erosion [6]. In the latter part of the 20th century, treatment regimens were expanded to include conventional csDMARDs such as methotrexate, hydroxychloroquine, and sulfasalazine, which remain an essential part of the therapeutic paradigm [7]. The progress achieved in drug development has contributed to the introduction, in the clinical armamentarium, of biological disease-modifying anti-rheumatic drugs (bDMARDs), which, to date, represent one of the most significant changes in the arthritis therapeutic landscape [8]. These genetically modified monoclonal antibodies and receptor constructs were specifically developed to target important inflammatory molecular mediators including tumor necrosis factor-alpha (TNF-α) [9], interleukin-1 (IL-1) and -6 (IL-6) [10] or surface substances implicated in activation of lymphocytes T [11] or B cell survival signals [12]. Similarly, the development of biosimilar drugs has further increased the availability and affordability of this new therapeutic approach by reaching the same targets. 

To date, in Italy, there are currently several bDMARDs available targeting the tumor necrosis factor (TNF): etanercept (ETN), infliximab (IFX), adalimumab (ADA), certolizumab (CZP), and golimumab (GOL). Besides these aforementioned drugs, other biologic agents working directly on different targets are available in the clinical armamentarium: abatacept (ABA), secukinumab (SEC), anakinra (ANA), rituximab (RTX), tocilizumab (TCZ), and sarilumab (SAR), ustekinumab (UST) [13]. Recently, targeted synthetic disease-modifying antirheumatic drugs (tsDMARDs) were discovered: apremilast, tofacitinib, baricitinib, and upadacitinib [14,15]. As reported, these new therapeutic approaches for IA have led to substantial improvements in patients’ management never achieved before with previous therapies [16].

Nevertheless, these drugs may be the source of serious adverse events (SAEs) and rare and unpredictable AEs. In fact, infections (including tuberculosis and opportunistic infections) [17,18], cardiovascular risk [19,20], skin cancers [21], neurological events [22], and hepatitis B reactivation [23] have been reported during bDMARD therapy. By virtue of this, the risk/benefit profile of these therapies, particularly for long-term treatments, deserves to be fully defined [24]. Unfortunately, these undesirable side effects are difficult to uncover in pre-marketing clinical trials because of several limits of clinical trials, such as short follow-up periods and rigorous inclusion criteria.

Active post-marketing surveillance plans (named pharmacovigilance) have significantly improved the detection and reporting of SAEs and unexpected AEs in the real world, providing important safety data for several treatments. Based on this background, the purpose of the current study is to present the preliminary results of a Regional Pharmacovigilance Program (Calabria, Italy) designed to improve reporting of adverse events (AEs) associated with biologics use in rheumatology. Although only a few centers have been involved in the current study, the consistent number of patients enrolled allowed us to obtain a suitable amount of clinical information, useful to improve the management of biological drugs.

## 2. Materials and Methods

### 2.1. Study Design 

The current investigation is a multicenter, prospective, observational cohort study based on the Calabria Biologics Pharmacovigilance Program (CBPP), a pharmacovigilance project aimed at the evaluation of the safety of biologics in clinical practice, including rheumatology units, in the Calabria region. The data presented in this study was obtained between 1 January 2016 and January 2022 from Rheumatology Outpatient Clinic, Azienda Ospedaliera “Mater Domini”, Catanzaro, Italy; Rheumatology Outpatient Clinic, Azienda Ospedaliera “Pugliese-Ciaccio”, Catanzaro, Italy and Rheumatology Unit, Azienda Ospedaliera “SS Annunziata”, Cosenza, Italy. A specialist monitor in clinical pharmacology with proper training in pharmacovigilance was assigned to each rheumatology unit. 

### 2.2. Study Population and Data Collection

All the consecutive patients were enrolled in the study if they satisfied the following inclusion criteria: (a) Age ≥ 16 years; (b) confirmed diagnosis of RA, PsA or AS according to ACR/EULAR classification criteria [25], CASPAR classification criteria [26] and ASAS criteria [27], respectively; and (c) treatment with bDMARD (both in monotherapy or co-administration with non-biologic drugs). For each patient, information on age, sex, clinical diagnosis, and smoking was collected. Clinical data including concomitant csDMARDs or corticosteroid therapies, current biologic therapy, the reason for discontinuation and switch/swap to another biologic agent, therapeutic failures, and the potential occurrence of AEs, were also acquired. Patients were considered to have discontinued therapy if the bDMARD had not been taken within the advised time or if the therapeutic plan was not renewed. Moreover, the term switch refers to the substitution of one biological agent with another with the same mechanism of action, while the term swap indicates the replacement of the drug with another one with a different action. A clinical pharmacologist and/or pharmacist recorded the potential AEs by completing, in the event of possible adverse drug reaction (ADR), the reporting form of the Italian Medicines Agency (AIFA). Concurrently, the clinical pharmacologist and/or pharmacist provided a detailed report, including the onset date and recovery, severity, and outcome, codifying the AE in agreement with the Medical Dictionary for Regulatory Activities (MedDRA^®^, The International Federation of Pharmaceutical Manufactures & Associations, IFPMA, Geneva, Switzerland) Preferred Term (PT) and System Organ Class (SOC) levels. An AE was defined as serious if it was life-threatening or fatal, required hospitalization (or prolonged existing hospitalization), resulted in persistent or significant disability or a congenital anomaly/birth defect, or was another medically critical condition. An encrypted code was used to maintain the patient’s data anonymity. Moreover, the Naranjo Adverse Probability Scale was applied to assess the relationship between AEs and drug treatment. 

### 2.3. Ethics Committee

Written informed consent was acquired from all participants at the time of enrolment. The local Ethics Committees of the Calabria Region approved the study protocol (Protocol No. 278/2015), and all investigations were performed in agreement with the 1964 Declaration of Helsinki and its amendments. 

### 2.4. Data Analysis

Descriptive statistical analyses were performed to assess enrolled patients’ clinical and demographic characteristics at the *index date*. Categorical variables are presented as frequencies and percentages, whereas continuous data are presented as mean ± standard deviation (SD) or median (25–75 percentile) as appropriate. Furthermore, the Pearson chi-square test was used to compare the number of switches/swaps between bDMARD groups. *p* < 0.05 was considered significant. IBM SPSS^®^ Statistics (IBM, Armonk, NY, USA), Version 26.0 was used to perform the statistical analysis.

## 3. Results

### 3.1. Characteristics of the Study Cohort

A total of 872 patients (536 females; mean age 61.4 ± 12 years) were enrolled in this pharmacovigilance program. Most patients had RA (371, 42.5%), followed by PsA (330, 37.8%) and AS (171, 19.6%). One-third of patients (295, 33.8%) were treated with ADA at the beginning of the study. ETN, IFX, and GOL were the other biologic agents most commonly administrated with 195 (22.4%), 104 (11.9%), and 89 (10.2%) patients, respectively. Furthermore, 348 (39.9%) patients received concomitant treatment with methotrexate (MTX) and 112 (12.8%) with corticosteroids (CCS). All demographic and clinical data are shown in Table 1. Overall, 729 patients (86.3%) did not develop AEs or SAEs, whereas 143 (16.4%) patients experienced at least one AE, of which 16 (1.8%) had at least one SAE. Data subdivided per drug are shown in Table 2.

### 3.2. Safety Profile and Treatment Failures

Switches/swaps that occurred during the observation are summarized in Table 3. Overall, 113 patients switched to another biologic with the same mechanism of action; 89 patients started a new biologic drug with a different mechanism of action, and 37 patients underwent both switch and swap, for a total of 173 switches and 156 swaps. Switches mostly occurred for primary/secondary inefficacy (136; 77.7%), whereas only 39 (22.3%) for the onset of an AE. Similarly, primary/secondary failure was the most frequent reason for the swaps (124, 79%), while AEs onset led to 33 (21%) swaps. The switch ADA/ETN (28; 16.2%), followed by the switch ETN/GOL (19; 11%), ADA/GOL (19; 11%), ETN/ADA (17, 9.8%) and the swap ADA/SEC and ADA/ABA (11; 7.1%, for both) were the most frequently performed in patients with a primary/secondary failure; while ADA/ETN (10; 5.8%), ETN/GOL (7; 4%), IFX/ADA (5; 2.9%), and ADA/ABA (5; 3.2%) occurred in patients with an AE. In addition, 61 subjects underwent at least a second switch/swap to another bDMARD; in detail, only 16 for the onset of an AE and 45 for inefficacy. Nonetheless, for 24 patients, a third switch/swap was necessary, mostly for therapeutic failure; three patients needed a fourth switch/swap (two for inefficacy and one for AE) and two patients had a fifth switch/swap both for lack of efficacy.

### 3.3. Switches and Swaps Frequencies among bDMARDs

A comparison between biological agents was conducted to identify potential differences. For switches (Table 4), only anti-TNFα agents were included, demonstrating statistically significant overall differences in the number of switches. Patients firstly treated with ADA underwent up to four switches, followed by IFX (up to three switches); patients treated with CZP, such as the first biologic drug, experienced fewer switches. In further detail, the pair differences were found between ADA vs. IFX (*p* = 0.005); ETN vs. GOL (*p* = 0.045); ETN vs. IFX (*p* = 0.017); GOL vs. IFX (*p* = 1.97 × 10^−4^). Table 5 shows the comparisons of the number of swaps that patients with a specific first biologic agent underwent during the whole observation. No significant differences were found among bDMARDs.

### 3.4. Characteristics of the Adverse Events

Overall, 190 AEs were reported in 143 patients that experienced at least one AE (Table 6). Specifically, 17 (8.9%) were SAEs occurring in 16 patients. According to the MedDRA^®^ SOC classification, the most frequently detected AEs were general disorders and administration site conditions (80; 42.1%), gastrointestinal (28; 14.7%), nervous system, skin, and subcutaneous tissue disorders (26; 13.6%), followed by respiratory, thoracic and mediastinal disorders (10; 5.3%). General and administration site conditions were mostly correlated to treatment with ADA (19; 23.8%); skin and subcutaneous tissue disorders with ABA (7; 26.9%); nervous system disorders with ADA7; 26.9%); respiratory, thoracic and mediastinal disorders with GOL (4; 40%) and gastrointestinal disorders with ADA (11; 39.3%). Concerning SAEs, the four cases of severe herpes virus infection were mainly related to ABA, followed by ADA and GOL. ADA was associated with a case of steatosis, a case of uveitis, and a case of pulmonary mass, while GOL was associated with tonsillar hypertrophy and syncope (one case each). Two cases of neutropenia and lymphocytosis were reported in patients treated with ABA and ETN. ABA was also associated with the onset of dyspnea and the occurrence of a case of umbilical hemorrhage. In contrast, a patient treated with ETN developed demyelination and another developed an influenza-like illness. A case of demyelination leukoencephalopathy developed in a patient who used SEC. Finally, a case of skin exfoliation was associated with UST. 

## 4. Discussion

Pharmacovigilance studies are crucial to detect the safety profile of bDMARDs overcoming different bias of randomized controlled trials, including short follow-up periods and strict inclusion criteria [14]. Accordingly, this multicenter study, aiming to assess the safety profile of biological drugs used in the rheumatology field, increases consciousness of AEs in a real-world setting, taking into account the data from earlier studies [15]. According to the literature data, our findings show that females are more frequently affected by rheumatologic disorders. This sex bias could be linked to estrogens since they are potent stimulators of autoimmunity [28,29,30]. Likewise, the median age of patients is in agreement with data previously reported [14]. Our findings on biologics reflect current clinical practice in using bDMARDs in the rheumatologic setting. According to the literature [31,32], ADA was the most commonly prescribed drug in our patients, followed by ETN and IFX. As previously observed [33,34], females had a higher significant onset of AEs than males. We have observed that the AE incidence was higher in women compared to men; this could be a consequence of variations in pharmacokinetic, immunological, and hormonal factors [35]. 

Less than a third of the patients underwent a switch/swap and the rate of the most common switches (ADA/ETN, ETN/GOL and ETN/ADA) was consistent with previous studies [36,37,38]. Overall, ADA was the biologic agent that most frequently led to switch and swap for both failure and AEs. However, this phenomenon is probably due to the high prevalence of patients treated with ADA. Moreover, about 40% of patients did not respond to a specific biologic and up to 20% were resistant to all currently available treatments. For this reason, our results are in line with previous data [39].

Our results showed that patients treated with ADA or IFX were more prone to switch towards another anti-TNFα. These findings have been described also in the inflammatory bowel disease setting, confirming that primary non-response to TNFα inhibitors is also associated with inferior response to second line agent such ustekinumab [40]. Based on these data, we can state that therapeutic management of chronic inflammatory conditions should be integrated with the analysis of the main molecular pathway involved in the pathogenesis of the disease [41]. 

Although there are few data available on the safety profile of biological agents in the rheumatologic field in the long-term, the described AEs in our study are consistent the Summaries of Product Characteristics (SPCs). The most common AEs were administration site conditions followed by gastrointestinal disorders, nervous system disorders and skin and subcutaneous tissue disorders (from mild to moderate). In particular, patients treated with ADA reported AEs related to administration site conditions mainly due to the high occurrence of injection-related reactions. Moreover, a higher risk of causing neurological disorders was described for ADA, which is the most involved among the TNFi with central nervous system disorders [22]. In our study, we also reported episodes of uveitis related to the use of ADA. This phenomenon might be considered an extra-articular symptom of inflammatory arthritis. Concerning skin disorders, it is well documented that anti-TNF drugs have a key role in the onset of dermatological symptoms, such as urticaria, erythema, and dermatitis, with a frequency ranging from 10% to 60% [42,43]. In addition, we also detected that ABA was more commonly linked to skin reactions and could be considered one of the risk factors for the occurrence of cutaneous AEs. In contrast to previously available literature data that demonstrated ABA’s safety profile, our data documented four cases of severe herpes virus infection compared to other bDMARDs leading to treatment withdrawal [18,44], as reported in another study. Furthermore, focusing on SAEs, our patients in treatment with ABA and ETN experienced severe neutropenia mostly associated with lymphocytosis. This phenomenon is probably related to the dysregulation of TNF ligands, such as T cells, caused by the direct action of the drug [45]. Our results reveal a better safety profile for ADA and IFX, while UST and SEC showed a significant probability of experiencing AEs or treatment failure. This trend was determined by considering the total number of AEs and the number of patients in treatment with the specific drug. For example, a total of 47 AEs were reported for ADA and 12 for IFX in a total of 295 and 104 patients, respectively; on the contrary 13 AEs/21 patients for UST and 19AEs/30 for SEC were described. 

## 5. Conclusions

From the very beginning, the Calabria Biologics Pharmacovigilance Program allowed us to collect informative data that over time have grown and are now suitable to be applied in clinical practice. The constant surveillance from the start date of biologic agent administration as well as the collaboration between specialists made it possible to monitor patients throughout the course of the disease. Our study reports data for about 800 patients affected by inflammatory arthritis and treated with biologics in a real-world setting during a six-year study period. The stated AEs, generally mild to moderate, were in line with those previously reported in the literature. The present data indicate that some safety risks, particularly those associated with long-term treatment, are still undiscovered. An aspect that should be considered concerns the origin of the data, provided only by patients from the Calabria region, which could not be representative of the entire Italian population. Indeed, the decision to treat a patient with a specific biologic agent may be influenced by distinct regional policies that control the prescription of bDMARDS. For this reason, multiregional studies could allow a better understanding of biologics safety profile. However, any data, even from limited post-marketing investigations, are crucial for establishing the exact relationship between AEs and a specific biologic drug.

## Figures and Tables

**Table 1:** Characteristics of the study population.

	Patients (*n* = 872)
Female sex, *n* (%)	536 (61.5)
Age, years	61.4 ± 12
Smoking, *n* (%)	81 (9.3)
Age first biologic agent, years	54.1 ± 11.9
Diagnosis	
Rheumatoid arthritis, *n* (%)	371 (42.5)
Psoriatic arthritis, *n* (%)	330 (37.8)
Spondylarthritis, *n* (%)	171 (19.6)
**First biological drugs prescribed**	
ADA, *n* (%)	295 (33.8)
ETN, *n* (%)	195 (22.4)
IFX, *n* (%)	104 (11.9)
GOL, *n* (%)	89 (10.2)
ABA, *n* (%)	76 (8.7)
TCZ, *n* (%)	41 (4.7)
SEC, *n* (%)	30 (3.4)
UST, *n* (%)	21 (2.4)
CZP, *n* (%)	15 (1.7)
IXE, *n* (%)	3 (0.3)
RTX, *n* (%)	2 (0.2)
GUS, *n* (%)	1 (0.1)
**Concurrent treatments**	
MTX, *n* (%)	348 (39.9)
CCSs, *n* (%)	112 (12.8)
NSAID, *n* (%)	30 (3.4)
HCQ, *n* (%)	23 (2.6)
LEF, *n* (%)	12 (1.4)
CyA, *n* (%)	7 (0.8)
SSZ, *n* (%)	3 (0.3)
Switch, *n* (%)	150 (17.2)
Swap, *n* (%)	126 (14.4)
**Adverse events**	
AEs, *n* (%)	143 (16.4)
SAEs, *n* (%)	16 (1.8)
**AEs**	
Female, *n* (%)	95 (17.7)
Male, *n* (%)	48 (14.3)
**SAEs**	
Female, *n* (%)	7 (1.3)
Male, *n* (%)	9 (2.7)

IFX, infliximab; ETN, etanercept; ADA, adalimumab; GOL, golimumab; TCZ, tocilizumab; CZP, certolizumab; UST, ustekinumab; SEC, secukinumab; ABA, abatacept; RTX, rituximab; IXE, ixekizumab, GUS, guselkumab; MTX, methotrexate; LEF, leflunomide; HCQ, idrossiclorochina; CyA, cyclosporin A; CCS, corticosteroids; SSZ, sulfasalazine; NSAIDs, Nonsteroidal anti-inflammatory drugs; AEs, adverse events; SAEs, serious adverse events.

**Table 2:** Characteristics of the study cohort per drugs.

	IFX (*n* = 104)	ETN (*n* = 195)	ADA (*n* = 295)	GOL (*n* = 89)	TCZ (*n* = 41)	CZP (*n* = 15)	UST (*n* = 21)	SEC (*n* = 30)	ABA (*n* = 76)
Female sex, *n* (%)	67 (64.4)	109(55.9)	177 (60.0)	53 (59.6)	36 (87.8)	10 (66.7)	14 (66.7)	18 (60)	50 (65.8)
Age, years	61.6 ± 11	62.2 ± 11	59.7 ± 13	60.2 ± 12	62.8 ± 11	55.0 ± 15	60.9 ± 11	60.3 ± 9	65.1 ± 10
Smoking, *n* (%)	15 (14.4)	26 (13.3)	19 (6.4)	2 (2.2)	7 (17.1)	4 (26.7)	1 (4.8)	2 (6.7)	6 (7.9)
Age at index date	54.3 ± 11	54.2 ± 11	53.1 ± 13	54.7 ± 12	53.9 ± 13	49.0 ± 14	54.5 ± 12	55.8 ± 9	57.7 ± 11
**Diagnosis**									
Rheumatoid arthritis, *n* (%)	45 (43.3)	69 (35.4)	110 (37.3)	21 (23.6)	41 (100)	8 (53.3)	-	-	76 (100)
Psoriatic arthritis, *n* (%)	29 (27.9)	90 (46.2)	129 (43.7)	34 (38.2)	-	6 (40.0)	16 (76.2)	21 (70)	-
Spondylarthritis, *n* (%)	30 (28.8)	36 (18.5)	56 (19.0)	34 (38.2)	-	1 (6.7)	5 (23.8)	9 (30)	-
**Concurrent** **treatments**									
MTX, *n* (%)	48 (46.2)	74 (37.9)	118 (40.0)	37 (41.6)	11 (26.8)	8 (53.3)	7 (33.3)	15 (50.0)	29 (38.2)
LEF, *n* (%)	4 (3.8)	2 (1.0)	4 (1.4)	-	-	-	-	1 (3.3)	1 (1.3)
HCQ, *n* (%)	2 (1.9)	9 (4.6)	5 (1.7)	3 (3.4)	1 (2.4)	-	1 (4.8)	1 (3.3)	1 (1.3)
CyA, *n* (%)	1 (1.0)	1 (0.5)	2 (0.7)	-	1 (2.4)	1 (6.7)	-	-	1 (1.3)
CCSs, *n* (%)	21 (20.2)	20 (10.3)	32 (10.8)	15 (16.9)	1 (2.4)	4 (26.7)	3 (14.3)	4 (13.3)	10 (13.2)
SSZ, *n* (%)	-	-	-	1 (1.1)	-	-	2 (9.5)	-	-
NSAID, *n* (%)	2 (1.9)	6 (3.1)	17 (5.8)	1 (1.1)	1 (2.4)	-	-	-	2 (2.6)
Switched, *n* (%)	39 (37.5)	42 (21.5)	58 (19.7)	9 (10.1)	-	1 (6.7)	-	1 (3.3)	-
Swap, *n* (%)	23 (22.1)	38 (19.5)	40 (13.6)	9 (10.1)	4 (9.8)	2 (13.3)	1 (4.8)	1 (3.3)	6 (7.9)
**Adverse events**									
AEs, *n* (%)	18 (17.3)	33 (16.9)	48 (16.3)	13 (14.6)	3 (7.3)	3 (20)	3 (14.3)	7 (23.3)	14 (18.4)
SAEs, *n* (%)	5 (4.8)	4 (2.1)	6 (2.0)	-	-	-	-	-	1 (1.3)

IFX, infliximab; ETN, etanercept; ADA, adalimumab; GOL, golimumab; TCZ, tocilizumab; CZP, certolizumab; UST, ustekinumab; SEC, secukinumab; ABA, abatacept; MTX, methotrexate; LEF, leflunomide; HCQ, idrossiclorochina; CyA, cyclosporin A; CCS, corticosteroids; SSZ, sulfasalazine; NSAIDs, Nonsteroidal anti-inflammatory drugs; AEs, adverse events; SAEs, serious adverse events.

**Table 3:** Summary of biologic drugs switch/swap.

Switch/Swap to													
**Switch/swap from**		**IFX**	**ETN**	**ADA**	**GOL**	**TCZ**	**CZP**	**UST**	**SEC**	**ABA**	**RTX**	**IXE**	**SAR**
	**IFX**		15 (1)	15 (5)	11 (2)	2 (1)	-	2	2	7 (3)	-	-	-
	**ETN**	3		20 (3)	26 (7)	5 (1)	2 (2)	9 (3)	11 (2)	13 (3)	-	-	-
	**ADA**	1	38 (10)		23 (4)	4	5 (1)	8 (1)	13 (2)	16 (5)	1	-	1
	**GOL**	3 (1)	3	6 (2)		2 (1)	1	4 (2)	5 (2)	5	-	1	1
	**TCZ**	-	4	-	1					5	-	-	-
	**CZP**	-	1	-	-	1			1	1	-	-	-
	**UST**	-	1	2	1	-	-		4		-	1	-
	**SEC**	-	-	1	-	-	-	1			-	-	-
	**ABA**	1	-	3 (1)	2 (1)	8 (1)	1	-	2 (1)		-	-	-
	**RTX**	-	-	1	-	1	-	-	-	1		-	-

Summary of biologic drugs switch/swap related to therapeutic failures and AEs (reported in brackets). The cells with green background indicate an impossible switch/swap from/to the same biologic drug. Abbreviations: IFX, infliximab; ETN, etanercept; ADA, adalimumab; GOL, golimumab; TCZ, tocilizumab; CZP, certolizumab; UST, ustekinumab; SEC, secukinumab; ABA, abatacept; RTX, rituximab. No switches to anakinra (not reported).

**Table 4:** No. of switches among anti-TFN bDMARDs.

N. Switch	ADA	CZP	ETN	GOL	IFX	*p*
**0**		14	153	80	65	0.02
**1**	51	1	38	7	33	
**2**	5	0	4	2	5	
**3**	1	0	0	0	1	
**4**	1	0	0	0	0	

ADA, adalimumab; CZP, certolizumab; ETN, etanercept; GOL, golimumab; IFX, infliximabADA vs. IFX: *p* = 0.005, ETN vs. GOL: *p* = 0.045, ETN vs. IFX: *p* = 0.017, GOL vs. IFX: *p* = 1.97 × 10^−4^.

**Table 5:** No. of swaps among bDMARDs.

N° Swap	ABA	ADA	CZP	ETN	GOL	IFX	SEC	TCZ	UST	RTX	IXE	GUS	*p*
**v**	70	255	13	157	80	81	29	37	20	0	3	1	0.096
**1**	5	31	2	31	9	16	1	4	1	2	0	0	
**2**	1	5	0	6	0	6	0	0	0	0	0	0	
**3**	0	4	0	1	0	1	0	0	0	0	0	0	

ABA, abatacept; ADA, adalimumab; CZP, certolizumab; ETN, etanercept; GOL, golimumab; IFX, infliximab; SEC, secukinumab; TCZ, tocilizumab; UST, ustekinumab; RTX, rituximab; IXE, ixekizumab; GUS, guselkumab.

**Table 6:** SOC-general disorders and administration site conditions.

Adverse Events	IFX (12)	ETN (25)	ADA (47)	GOL (28)	TCZ (12)	CZP (7)	UST (13)	SEC (19)	ABA (27)
**Pyrexia**		1		1					1
**Administration site reactions**		8	6	2	3	3	2	2	2
**Asthenia**	2	4	11	2		1		4	4
**Chest pain**		1							1
**Tiredness**	4		2	5	1	1	2	1	3
**TOT SOC (80)**	6	14	19	10	4	5	4	7	11
***SOC–Skin and subcutaneous tissue disorders***									
**Rash**			1	2					2
**Pruritus**	2	1	1				1		1
**Erythema**		1					1	1	2
**Sweating**		1	1	1	1			1	1
**Urticaria**					1				1
**Alopecia**	1							1	
**TOT SOC (26)**	3	3	3	3	2	-	2	3	7
***SOC—Cardiac disorders***									
**Tachycardia**									
**TOT SOC (0)**	-	-	-	-	-	-	-	-	-
***SOC—Vascular disorders***									
**Hypertension**	2								
**TOT SOC (2)**	2	-	-	-	-	-	-	-	-
***SOC—Nervous system disorders***									
**Headache**		2	5	5		2	1	2	2
**Drowsiness**	1		1						
**Confused state**		1	1	1					
**Demyelination**		1						1	
**TOT SOC (26)**	1	4	7	6	-	2	1	3	2
***SOC—Infections and infestations***									
**Herpes virus infection**			1	1					2
**TOT SOC (4)**	-	-	1	1	-	-	-	-	2
***SOC—Respiratory, thoracic and mediastinal disorders***									
**Dyspnea**					1				1
**Cough**			1	1					
**Tonsillitis**				1					1
**Sore throat**				2				1	
**Rhinitis**									1
**TOT SOC (10)**	-	-	1	4	1	-	-	1	3
***SOC—Blood and lymphatic system disorders***									
**Anemia**							1		1
**TOT SOC (2)**	-	-	-	-	-	-	1	-	1
***SOC—Gastrointestinal disorders***									
**Nausea**		2	10	2	1		2	2	1
**Dyspepsia**			1		1			2	
**Diarrhea**		1		1			1	1	
**TOT SOC (28)**	-	3	11	3	2	-	3	5	1
***SOC—Immune system disorders***									
**Uveitis**			1						
**TOT SOC (1)**	-	-	1	-	-	-	-	-	-
***SOC—Eye disorders***									
**Vision disorders**		1	1		1				
**TOT SOC (3)**	-	1	1	-	1	-	-	-	-
***SOC—Ear and labyrinth disorders***									
**Vertigo**			2		2		2		
**TOT SOC (6)**	-	-	2	-	2	-	2	-	-
***SOC—Musculoskeletal and connective tissue disorders***									
**Myalgia**				1					
**TOT SOC (1)**	-	-	-	1	-	-	-	-	-
***SOC-Hepatobiliary disorders***									
**Hepatic steatosis**			1						
**TOT SOC (1)**	-	-	1	-	-	-	-	-	-

## Data Availability

Not applicable.

## References

[1] Medrado L.N., Mendonça M.L.M., Budib M.B., Oliveira-Junior S.A., Martinez P.F. (2022). Effectiveness of aquatic exercise in the treatment of inflammatory arthritis: Systematic review. Rheumatol. Int..

[2] Rodrigues R., Ferraz R.B., Kurimori C.O., Guedes L.K., Lima F.R., de Sá-Pinto A.L., Gualano B., Roschel H. (2020). Low-Load Resistance Training With Blood-Flow Restriction in Relation to Muscle Function, Mass, and Functionality in Women With Rheumatoid Arthritis. Arthritis Care Res..

[3] Zhang Y., Zeng C., Li H., Yang T., Deng Z.H., Yang Y., Ding X., Xie D.X., Wang Y.L., Lei G.H. (2015). Relationship between cigarette smoking and radiographic knee osteoarthritis in Chinese population: A cross-sectional study. Rheumatol. Int..

[4] Abtahi S., Cordtz R., Dreyer L., Driessen J.H.M., Boonen A., Burden A.M. (2022). Biological Disease-Modifying Antirheumatic Drugs and Osteoporotic Fracture Risk in Patients with Rheumatoid Arthritis: A Danish Cohort Study. Am. J. Med..

[5] Zhu Y., Zhao T., Liu M., Wang S., Liu S., Yang Y., Yang Y., Nan Y., Huang Q., Ai K. (2022). Rheumatoid arthritis microenvironment insights into treatment effect of nanomaterials. Nano Today.

[6] Feng X., Wang X. (2018). Comparison of the efficacy and safety of non-steroidal anti-inflammatory drugs for patients with primary dysmenorrhea: A network meta-analysis. Mol. Pain.

[7] Burmester G.R., Bijlsma J.W.J., Cutolo M., McInnes I.B. (2017). Managing rheumatic and musculoskeletal diseases—Past, present and future. Nat. Rev. Rheumatol..

[8] Harrington R., Al Nokhatha S.A., Conway R. (2020). JAK Inhibitors in Rheumatoid Arthritis: An Evidence-Based Review on the Emerging Clinical Data. J. Inflamm. Res..

[9] Palladino M.A., Bahjat F.R., Theodorakis E.A., Moldawer L.L. (2003). Anti-TNF-alpha therapies: The next generation. Nat. Rev. Drug Discov..

[10] Dayer J.M., Oliviero F., Punzi L. (2017). A Brief History of IL-1 and IL-1 Ra in Rheumatology. Front. Pharmacol..

[11] Okuda Y. (2008). Review of tocilizumab in the treatment of rheumatoid arthritis. Biologics.

[12] Schiff M. (2011). Abatacept treatment for rheumatoid arthritis. Rheumatology.

[13] Lopez-Olivo M.A., Amezaga Urruela M., Mcgahan L., Pollono E.N., Suarez-Almazor M.E. (2015). Rituximab for rheumatoid arthritis. Cochrane Database Syst. Rev..

[14] Barbieri M.A., Cicala G., Cutroneo P.M., Gerratana E., Palleria C., De Sarro C., Vero A., Iannone L., Manti A., Russo E. (2020). Safety Profile of Biologics Used in Rheumatology: An Italian Prospective Pharmacovigilance Study. J. Clin. Med..

[15] Palleria C., Iannone L., Leporini C., Citraro R., Manti A., Caminiti M., Gigliotti P., Grembiale R.D., L’Andolina M., Muccari G. (2018). Implementing a simple pharmacovigilance program to improve reporting of adverse events associated with biologic therapy in rheumatology: Preliminary results from the Calabria Biologics Pharmacovigilance Program (CBPP). PLoS ONE.

[16] Rønholt K., Iversen L. (2017). Old and New Biological Therapies for Psoriasis. Int. J. Mol. Sci..

[17] Rutherford A.I., Patarata E., Subesinghe S., Hyrich K.L., Galloway J.B. (2018). Opportunistic infections in rheumatoid arthritis patients exposed to biologic therapy: Results from the British Society for Rheumatology Biologics Register for Rheumatoid Arthritis. Rheumatology.

[18] Atzeni F., Sarzi-Puttini P., Botsios C., Carletto A., Cipriani P., Favalli E.G., Frati E., Foschi V., Gasparini S., Giardina A.R. (2012). Long-term anti-TNF therapy and the risk of serious infections in a cohort of patients with rheumatoid arthritis: Comparison of adalimumab, etanercept and infliximab in the GISEA registry. Autoimmun. Rev..

[19] Zhang J., Xie F., Yun H., Chen L., Muntner P., Levitan E.B., Safford M.M., Kent S.T., Osterman M.T., Lewis J.D. (2016). Comparative effects of biologics on cardiovascular risk among older patients with rheumatoid arthritis. Ann. Rheum. Dis..

[20] Atzeni F., Svenungsson E., Nurmohamed M.T. (2019). Do DMARDs and biologic agents protect from cardiovascular disease in patients with inflammatory arthropathies?. Autoimmun. Rev..

[21] Marino F., Nucera V., Gerratana E., Fiorenza A., Sangari D., Miceli G., Masala I.F., Atzeni F. (2020). Cancer risk and tumour necrosis factor inhibitors in patients with inflammatory arthritis. Pharmacol. Res..

[22] Atzeni F., Talotta R., Masala I.F., Gerardi M.C., Casale R., Sarzi-Puttini P. (2018). Central nervous system involvement in rheumatoid arthritis patients and the potential implications of using biological agents. Best Pract. Res. Clin. Rheumatol..

[23] Lin T.C., Yoshida K., Tedeschi S.K., de Abreu M.M., Hashemi N., Solomon D.H. (2018). Risk of Hepatitis B Virus Reactivation in Patients With Inflammatory Arthritis Receiving Disease-Modifying Antirheumatic Drugs: A Systematic Review and Meta-Analysis. Arthritis Care Res..

[24] Smolen J.S., Landewé R.B.M., Bijlsma J.W.J., Burmester G.R., Dougados M., Kerschbaumer A., McInnes I.B., Sepriano A., Van Vollenhoven R.F., De Wit M. (2020). EULAR recommendations for the management of rheumatoid arthritis with synthetic and biological disease-modifying antirheumatic drugs: 2019 update. Ann. Rheum. Dis..

[25] Aletaha D., Neogi T., Silman A.J., Funovits J., Felson D.T., Bingham C.O., Birnbaum N.S., Burmester G.R., Bykerk V.P., Cohen M.D. (2010). 2010 Rheumatoid arthritis classification criteria: An American College of Rheumatology/European League Against Rheumatism collaborative initiative. Arthritis Rheum..

[26] Taylor W., Gladman D., Helliwell P., Marchesoni A., Mease P., Mielants H. (2006). Classification criteria for psoriatic arthritis: Development of new criteria from a large international study. Arthritis Rheum..

[27] Rudwaleit M., Van Der Heijde D., Landewé R., Listing J., Akkoc N., Brandt J., Braun J., Chou C.T., Collantes-Estevez E., Dougados M. (2009). The development of Assessment of SpondyloArthritis international Society classification criteria for axial spondyloarthritis (part II): Validation and final selection. Ann. Rheum. Dis..

[28] Ortona E., Pierdominici M., Maseli A., Veroni C., Aloisi F., Shoenfeld Y. (2016). Sex-based differences in autoimmune diseases. Ann. Ist. Super. Sanita.

[29] Nas K., Capkin E., Dagli A.Z., Cevik R., Kilic E., Kilic G., Karkucak M., Durmus B., Ozgocmen S. (2017). Gender specific differences in patients with psoriatic arthritis. Mod. Rheumatol..

[30] Palazzo C., Ravaud J.F., Papelard A., Ravaud P., Poiraudeau S. (2014). The burden of musculoskeletal conditions. PLoS ONE.

[31] Esposti L.D., Perrone V., Sangiorgi D., Buda S., Andretta M., Rossini M., Girolomoni G. (2018). Analysis of drug utilization and health care resource consumption in patients with psoriasis and psoriatic arthritis before and after treatment with biological therapies. Biologics.

[32] Stamm T.A., Reichardt B., Zwerina J., Ritschl V., Nell-Duxneuner V. (2018). Use of biological disease modifying antirheumatic drugs in rheumatoid arthritis in Austria from 2008 to 2011: A retrospective analysis of 72% of the population. Wien. Klin. Wochenschr..

[33] Scavone C., Sportiello L., Sullo M.G., Ferrajolo C., Ruggiero R., Sessa M., Berrino P.M., di Mauro G., Berrino L., Rossi F. (2017). Safety Profile of Anticancer and Immune-Modulating Biotech Drugs Used in a Real World Setting in Campania Region (Italy): BIO-Cam Observational Study. Front. Pharmacol..

[34] Rusman T., Ten Wolde S., Euser S.M., van der Ploeg T., van Hall O., van der Horst-Bruinsma I.E. (2018). Gender differences in retention rate of tumor necrosis factor alpha inhibitor treatment in ankylosing spondylitis: A retrospective cohort study in daily practice. Int. J. Rheum. Dis..

[35] Rademaker M. (2001). Do women have more adverse drug reactions?. Am. J. Clin. Dermatol..

[36] Santoleri F., Romagnoli A., Costantini A. (2020). Adalimumab and etanercept adherence, persistence and switch in the treatment of psoriatic arthritis: 10-year real-life analysis. Expert Opin. Drug Saf..

[37] Caporali R., Sarzi-Puttini P., Atzeni F., Gorla R., Filippini M., Marchesoni A., Favalli E.G., Bobbio-Pallavicini F., Montecucco C. (2010). Switching TNF-alpha antagonists in rheumatoid arthritis: The experience of the LORHEN registry. Autoimmun. Rev..

[38] Bonafede M., Fox K.M., Watson C., Princic N., Gandra S.R. (2012). Treatment patterns in the first year after initiating tumor necrosis factor blockers in real-world settings. Adv. Ther..

[39] Buch M.H. (2019). Response to “Correspondence to viewpoint ‘Defining refractory rheumatoid arthritis’ by Buch” by Roodenrijs et al. Ann. Rheum. Dis..

[40] Singh S., George J., Boland B.S., Vande Casteele N., Sandborn W.J. (2018). Primary Non-Response to Tumor Necrosis Factor Antagonists is Associated with Inferior Response to Second-line Biologics in Patients with Inflammatory Bowel Diseases: A Systematic Review and Meta-analysis. J. Crohns. Colitis.

[41] Rivellese F., Surace A.E.A., Goldmann K., Sciacca E., Çubuk C., Giorli G., John C.R., Nerviani A., Fossati-Jimack L., Thorborn G. (2022). Rituximab versus tocilizumab in rheumatoid arthritis: Synovial biopsy-based biomarker analysis of the phase 4 R4RA randomized trial. Nat. Med..

[42] Lee H.H., Song I.H., Friedrich M., Gauliard A., Detert J., Röwert J., Audring H., Kary S., Burmester G.R., Sterry W. (2007). Cutaneous side-effects in patients with rheumatic diseases during application of tumour necrosis factor-alpha antagonists. Br. J. Dermatol..

[43] Flendrie M., Vissers W.H.P.M., Creemers M.C.W., de Jong E.M.G.J., van de Kerkhof P.C.M., van Riel P.L.C.M. (2005). Dermatological conditions during TNF-alpha-blocking therapy in patients with rheumatoid arthritis: A prospective study. Arthritis Res. Ther..

[44] Cassone G., Manfredi A., Atzeni F., Venerito V., Vacchi C., Picerno V., Furini F., Erre G.L., Tomietto P., Fedele A.L. (2020). Safety of Abatacept in Italian Patients with Rheumatoid Arthritis and Interstitial Lung Disease: A Multicenter Retrospective Study. J. Clin. Med..

[45] Ursini F., Naty S., Bruno C., Calabria M., Spagnolo F., Grembiale R.D. (2010). CD4+ T-cells lymphocytosis and reduction of neutrophils during treatment with adalimumab: Challenge and dechallenge study. Clin. Immunol..

